# Supplementary material for: Lowland plant arrival in alpine ecosystems facilitates a decrease in soil carbon content under experimental climate warming
Source: eLife. 2022 May 12;11:e78555. doi: 10.7554/eLife.78555 (PMC9191888; doi:10.7554/eLife.78555)
Supplement: Supplementary file 1. — LR: likelihood ratio. [file elife-78555-supp1.docx]

| **Response** | **Treatment** | | **Region** | | **Treatment × Region** | |  |
| --- | --- | --- | --- | --- | --- | --- | --- |
|  | LR | *P* | LR | *P* | LR | *P* | N |
| Soil carbon content | 23.16 | < 0.0001 | 34.53 | < 0.0001 | 4.64 | 0.0984 | 58 |
| Net ecosystem CO_2_ exchange | 23.26 | < 0.0001 | 40.76 | < 0.0001 | 6.43 | 0.0401 | 266 |
| Gross primary production | 58.99 | < 0.0001 | 50.64 | < 0.0001 | 2.78 | 0.2491 | 264 |
| Ecosystem respiration | 49.61 | < 0.0001 | 13.08 | 0.0045 | 12.98 | 0.0015 | 268 |
| Microbial biomass C | 33.52 | < 0.0001 | 13.64 | 0.0034 | 5.35 | 0.0688 | 58 |
| Microbial growth (per gram soil) | 9.33 | 0.0094 | - | - | - | - | 27 |
| Microbial respiration (per gram soil) | 1.20 | 0.5500 | - | - | - | - | 28 |
| Microbial growth (biomass-specific) | 8.32 | 0.0156 | - | - | - | - | 27 |
| Microbial Respiration (biomass-specific) | 6.54 | 0.0381 | - | - | - | - | 28 |
| Microbial carbon use efficiency | 15.68 | 0.0004 | - | - | - | - | 58 |
