# Supplementary material for: Lowland plant arrival in alpine ecosystems facilitates a decrease in soil carbon content under experimental climate warming
Source: eLife. 2022 May 12;11:e78555. doi: 10.7554/eLife.78555 (PMC9191888; doi:10.7554/eLife.78555)
Supplement: Supplementary file 2. — LR: likelihood ratio. [file elife-78555-supp2.docx]

| **Response variable** | **LR** | **N** | ***P*** |
| --- | --- | --- | --- |
| Pot-level plant traits |  |  |  |
| Aboveground biomass | 4.85 | 19 | 0.0277 |
| Belowground biomass | 16.31 | 19 | 0.0001 |
| Root-to-shoot ratio | 7.92 | 19 | 0.0049 |
| Specific leaf area (SLA) | 0.03 | 18 | 0.8605 |
| Maximal photosynthetic capacity (A_max_) | 4.98 | 19 | 0.0257 |
| Maximal stomatal conductance (g_s_) | 14.37 | 20 | 0.0002 |
|  |  |  |  |
| Soil variables |  |  |  |
| Soil pore water absorbance (a_350_) | 24.84 | 19 | < 0.0001 |
| Soil pore water total fluorescence (F_tot_) | 27.72 | 19 | < 0.0001 |
| Soil pore water fluorescence index (FI) | 9.55 | 19 | 0.0020 |
| Microbial biomass carbon | 0.16 | 19 | 0.6932 |
| Fast-decaying soil carbon pool size | 3.99 | 16 | 0.0458 |
| Fast-decaying soil carbon pool decay rate | 0.23 | 16 | 0.6306 |
| Soil DOM C1 (protein-like) | 21.39 | 19 | < 0.0001 |
| Soil DOM C2 (protein-like) | 10.42 | 19 | 0.0012 |
| Soil DOM C3 (humic-like) | 15.65 | 19 | 0.0001 |
| Soil DOM C4 (humic-like) | 9.60 | 19 | 0.0019 |
| Soil DOM C5 (humic-like) | 17.39 | 19 | < 0.0001 |
| Soil DOM C6 (fulvic acid-like) | 16.50 | 19 | < 0.0001 |
