# Supplementary material for: Lowland plant arrival in alpine ecosystems facilitates a decrease in soil carbon content under experimental climate warming
Source: eLife. 2022 May 12;11:e78555. doi: 10.7554/eLife.78555 (PMC9191888; doi:10.7554/eLife.78555)
Supplement: Supplementary file 3. [file elife-78555-supp3.docx]

| **Explanatory variable** | **Linear model test statistics** | | |
| --- | --- | --- | --- |
|  | F | d.f. | *P* |
| Western Alps experiment |  |  |  |
| *Achillea millefolium* | 0.17 | 1,4 | 0.7007 |
| *Bellis perennis* | 0.40 | 1,4 | 0.5634 |
| *Bromus erectus^*^* | - | - | - |
| *Dactylis glomerata^*^* | - | - | - |
| *Medicago lupulina^*^* | - | - | - |
| *Plantago media* | 1.19 | 1,4 | 0.3364 |
| *Salvia pratensis* | 0.10 | 1,4 | 0.7684 |
|  |  |  |  |
| Central Alps experiment |  |  |  |
| *Brachypodium pinnatum* | 3.70 | 1,8 | 0.3053 |
| *Carex flacca* | 28.24 | 1,8 | 0.1184 |
| *Carum carvi* | 5.94 | 1,8 | 0.2479 |
| *Dactylis glomerata^*^* | - | - | - |
| *Hypericum perforatum* | 10.11 | 1,8 | 0.1939 |
| *Plantago lanceolata^*^* | - | - | - |
| *Primula veris* | 6.41 | 1,8 | 0.2395 |
| *Ranunculus bulbosus* | 40.38 | 1,8 | 0.0994 |
| *Salvia pratensis* | 0.08 | 1,8 | 0.8281 |
| *Silene vulgaris* | 36.12 | 1,8 | 0.1050 |
| *Trifolium montanum^*^* | - | - | - |
| *Viola hirta^*^* | - | - | - |

*^*^ NB: species not tested, present in < 3 plots*
