# Supplementary material for: Lowland plant arrival in alpine ecosystems facilitates a decrease in soil carbon content under experimental climate warming
Source: eLife. 2022 May 12;11:e78555. doi: 10.7554/eLife.78555 (PMC9191888; doi:10.7554/eLife.78555)
Supplement: Supplementary file 4. [file elife-78555-supp4.docx]

| Name | Excitation  λ (nm) | Emission  Λ (nm) | General description^†^ | Peak name^*^ |
| --- | --- | --- | --- | --- |
| C1 | 275 | 332 | Protein-like (tryptophan) | T |
| C2 | 270 | 310 | Protein-like (tyrosine) | B |
| C3 | 340 | 434 | Humic-like | A/C |
| C4 | 305 | 424 | Microbial humic-like | M |
| C5 | 375 | 452 | Humic-like | C |
| C6 | < 250 (400) | 506 | Soil fluvic acid-like | C+ |

^*^ Ref 57; ^†^ Ref 56
